# Genetic regulation of sperm DNA methylation in cattle through meQTL mapping

**DOI:** 10.1186/s12864-025-11934-x

**Published:** 2025-08-22

**Authors:** Corentin Fouéré, Valentin Costes, Chris Hozé, Amrita Raja Ravi Shankar, Florian Besnard, Gabriel Costa Monteiro Moreira, Valentin Sorin, Chrystelle Le Danvic, Aurélie Chaulot-Talmon, Francesca Ali, Marie Christine Deloche, Aurélie Bonnet, Eliaou Sellem, Hélène Jammes, Sébastien Fritz, Mekki Boussaha, Didier Boichard, Hélène Kiefer, Marie-Pierre Sanchez

**Affiliations:** 1 Eliance, 149 Rue de Bercy, Paris, 75012 France; 2 Université Paris-Saclay, INRAE, AgroParisTech, GABI, Jouy-en-Josas, 78352 France; 3 Université Paris-Saclay, UVSQ, INRAE, BREED, Jouy-en-Josas, 78350 France; 4 Ecole Nationale Vétérinaire d’Alfort, BREED, Maisons-Alfort, 94700 France

**Keywords:** MeQTL mapping, GWAS, DNA methylation, Cattle, Semen

## Abstract

**Background:**

DNA methylation (DNAm) plays an important functional role and is influenced by genetic variants known as methylation QTLs (meQTLs). The majority of meQTL studies have been conducted in human blood. Despite its unique landscape, the genetic regulation of sperm DNAm remains largely unexplored. In this study, we leveraged DNAm measured in sperm from 405 Holstein bulls using reduced representation bisulfite sequencing (RRBS) and performed sequence-level genome-wide association studies for 166,985 variable CpGs (s.d. >5%). We reported heritability estimates and have mapped both *cis-*meQTLs and *trans-*meQTLs.

**Results:**

Heritability estimates ranged from 0 to 1 and averaged 0.26 across all selected CpGs, with 76% of estimates above 0.1. The meQTL mapping revealed that 32.9% of the CpGs had a *cis-*meQTL, 3.6% had a *trans-*meQTL and 1.0% had both *cis-* and *trans-*meQTLs. The *cis-*CpGs were located on average 261 kb (absolute mean) from their *cis-*meQTL top SNPs (defined by the most significant association). MeQTLs were enriched in featured genomic annotations, including regions surrounding transcription start sites and ATAC-seq peaks. We also identified spurious *trans-*associations by analyzing data across multiple genome assemblies, including the construction of a partial pangenome. Additionally, eight *trans-*meQTL hotspots, defined as variants associated with at least 30 *trans-*CpGs, were identified and overlapped with genes involved in epigenetic regulation. Using peripheral blood mononuclear cell DNAm from 54 out of the 405 bulls, we did not observe a similar effect of the *trans-*meQTL hotspots to that one observed in sperm.

**Conclusions:**

For the first time, meQTLs have been detected and characterized in bovine sperm, contributing to a better understanding of the transmission of paternally inherited DNAm marks. These findings provide useful information for further research aimed at integrating epigenetic information into the prediction of performance traits.

**Supplementary Information:**

The online version contains supplementary material available at 10.1186/s12864-025-11934-x.

## Background

DNA methylation (DNAm) in eukaryotes corresponds to the addition of a methyl group to the 5-carbon position of the cytosine pyrimidine ring mainly within CpG dinucleotides, resulting in 5-methylcytosine [1]. DNAm is a key epigenetic mark that regulates gene expression, is involved in cell differentiation [2] and maintenance of cell identity [3], and can lead to phenotypic changes in response to environmental exposures [4].

Large-scale epigenotyping (e.g., DNAm arrays) providing DNAm for several thousands to several hundreds of thousands of CpGs, has facilitated the study of genetic influence on DNAm, mainly in humans. The heritability reported for these sites is predominantly low to moderate, ranging between 0.1 and 0.3 [5, 6]. Genome-wide association studies (GWAS) have significantly advanced our understanding of gene regulation by mapping methylation quantitative trait loci (meQTL) that influence DNAm either at nearby (*cis*; within 1 Mb) or distant (*trans*; >1 Mb or interchromosomal) genomic locations [5–9]. The majority of meQTL are *cis*-acting [6, 8–10] and the proportion of reported *trans*-meQTL varies depending on the statistical power and significance threshold considered [5]. A recent study, based on blood DNAm from the 450 K array of 27,750 European individuals, reported meQTL associated with 45% of CpGs, out of which 8.5% were *trans*-meQTLs [8]. To further explore the biological links between gene regulation, environment, and phenotype, several studies have integrated the identification of meQTL into the study of complex diseases, e.g., by combining meQTL with expression QTL (eQTL) [10–12]. However, only a few studies have investigated DNAm genetics in farm animal species [13–15] and notably, their heritability’s estimates for DNAm measured in blood, are comparable to those found in human studies (i.e., 0.2 for global DNAm in sheep [13] and ranging from 0.18 to 0.72 across 34,324 CpGs using the HorvathMammalMethylChip40 array in cattle [15]).

Germ cells undergo extensive epigenetic reprogramming during their development, involving the erasure and re-establishment of their methylome [16]. Similar to other mammalian species, bovine spermatozoa display distinct methylation profile compared to somatic cells [17, 18]. This unique remodeling of DNAm landscape plays a crucial role in biological processes related to fertilization, post-fertilization reprogramming and the acquisition of totipotency [16]. As most studies have focused on blood DNAm, the genetic impacts of DNAm in sperm remains poorly understood, highlighting a need for further investigation.

Disentangling genetic and environmental factors affecting DNAm is of particular interest in sperm as it contains epigenetic marks that could be transmitted to the next generation. In the present study, we aimed to characterize the genetic architecture of DNAm determined through Reduced Representation Bisulfite Sequencing (RRBS), in sperm of 405 Holstein bulls. We estimated heritability and identified both *cis-* and *trans-*meQTLs associated with CpGs displaying variable DNAm, and further investigated these meQTL and their associated CpGs.

## Methods

### Animals, samples and genotypes

Semen samples were collected from 405 marketed Holstein bulls born between 2001 and 2021. The samples were obtained from frozen straws stored at −196 °C for on-farm artificial insemination. At the time of semen collection, the bulls ranged in age from 10 months to 7.3 years. Depending on the bulls, either pools of ejaculates or individual ejaculates (from one to nine samples per bull) were assessed. For 54 out of the 405 bulls, blood samples were collected from 15 months to 21 months of age. Peripheral blood mononuclear cells (PBMCs) were isolated from blood by centrifugation using a Ficoll gradient. The metadata for the samples can be found in Supplementary Table 1. All biological samples were collected with the informed consent of the breeders to whom the bulls belonged.

All bulls were genotyped using medium density SNP arrays (including the 50 K SNP Beadchip (Illumina, San Diego, CA, USA) and EuroGMD Beadchip (https://www.eurogenomics.com/actualites/the-eurog-md:-a-unique-genotyping-microarray-for-cattle-.html, Illumina, San Diego, CA, USA)). After quality control (as previously described by Sanchez et al. [19]) and imputation to the EuroGMD Beadchip, 53,469 autosomal SNPs were retained. The EuroGMD genotypes were then imputed up to whole-genome sequence level in a two-step approach: from EuroGMD to 777 K SNPs (BovineHD BeadChip; Illumina, San Diego, CA, USA) using FImpute [20], and then from 777 K SNPs to whole-genome sequence using Minimac [21], as described in Sanchez et al. [22]. We selected only variants with a Minimac coefficient of determination (r^2^) above 0.3. Finally, following the recommendations of Sahana et al., [23] we used a 3% minor allele frequency (MAF) threshold to ensure at least 20 copies of the rare allele, yielding a final dataset of 12,930,389 variants.

### DNA methylation

The equivalent of one straw of bull semen was used for DNA extraction (about 20 million spermatozoa). After thawing at 37 °C, the semen was washed with phosphate buffer saline (PBS) to remove the extender, washed again with H_2_O to lysate somatic cells, and incubated overnight at 55 °C in 200 µl lysis buffer (10 mM Tris-HCl pH 7.5, 25 mM EDTA, 1% SDS, 75 mM NaCl, 50 mM dithiothreitol (DTT) and 0.5 µg glycogen) in the presence of 0.2 mg/ml proteinase K. After incubation with 25 µg/ml RNAse A for 1 h at 37 °C, genomic DNA was extracted twice using phenol: chloroform (1:1) and chloroform, then ethanol precipitated and washed. The dried pellet was re-suspended in TE buffer (10 mM Tris HCl pH7.5, 2 mM EDTA) and the DNA concentration was measured using a Qubit 2.0 Fluorometer (Invitrogen). A similar procedure was applied to extract DNA from PBMCs, except that DTT was omitted. The integrity of DNA was checked using agarose electrophoresis.

For one subset of sperm samples (see Supplementary Table 1 for the detailed list), the RRBS libraries were constructed using a semi-automated procedure described in details in Costes et al. [24]. The other libraries were constructed by Integragen SA using a similar protocol based on MspI and the selection of genomic DNA fragments spanning from 40 to 290 bp. Sequencing was carried-out by Integragen SA to produce 75 bp paired-end reads (HiSeq4000, Illumina) or 100 bp paired-end reads (NovaSeq6000 and NovaSeq X Plus, Illumina).

On average, sequencing generated around 48 million read pairs per library. The sequences displayed the expected nucleotide composition based on MspI digestion and bisulfite conversion according to FastQC quality control. Subsequent quality checks and trimming were carried out using TrimGalore v0.4.4 [25] which removed adapter sequences, poor quality bases (Phred score below 20) and reads shorter than 20 nucleotides. The average bisulfite conversion rate was 99% and was estimated from unmethylated cytosine added in vitro during the end-repair step. High quality reads were aligned on the bovine reference genome (ARS-UCD1.2) using Bismark v0.20.0 [26] in the default mode with hisat2. After alignment, per sample, only CpGs covered by at least 10 uniquely mapped reads were retained (median = 27 reads per CpG). For these CpGs, a methylation percentage was calculated from Bismark methylation calling (number of reads with “C” × 100)/(number of reads with “C” + number of reads with “T”).

For the bulls with several RRBS samples analyzed, we computed the average DNAm percentage by CpG weighted by the number of reads in each sample. We then retained all CpGs with methylation information for at least 80% of the bulls. Any CpG that overlapped with known variants [27] was discarded. We then selected CpGs with a DNAm standard deviation (s.d.) >5% and corrected the methylation levels at each site for age at collection using four classes (< 500 d; [500d; 750 d];]750d; 1000 d]; >1000d).

### Genetic analysis

#### meQTL mapping

We performed single-trait analyses between the 12,930,389 variants and the 166,985 CpGs. The software GCTA (version 1.93) [28] with the *mlma* option was used to test the effect of each variant in the following mixed linear model:

1 $$\:\varvec{y}=\varvec{1}\mu\:+\varvec{x}b+\varvec{u}+\varvec{e}$$

where $\:\varvec{y}$ is the vector of methylation values for a given CpG, **1** is a vector of 1 s, $\:\mu\:$ is the overall mean, $\:b$ is the additive fixed effect of the variant to be tested for association, $\:\varvec{x}$ is the vector of SNP genotypes coded as 0, 1 or 2, $\:\varvec{u}\:\sim\:N(0,\varvec{G}{\sigma\:}_{u}^{2})$ is the vector of random polygenic effects, with $\:\varvec{G}$ the genomic relationship matrix (GRM), calculated using the EuroGMD SNPs genotypes, and $\:{\sigma\:}_{u}^{2}$ is the polygenic variance, estimated based on the null model ($\:\varvec{y}=\varvec{1}\mu\:+\varvec{u}+\varvec{e}$) and then fixed while testing for the association between each variant and the given CpG. Genomic inflation factor ($\:{\lambda\:}_{gc}$) was computed for each GWAS (from the median *P*-value). *P*-values were then adjusted for their corresponding $\:{\lambda\:}_{gc}$. MeQTLs were classified as *cis* if the distance between the CpG and the variant was within 1 Mb and as *trans* if this distance was greater than 2 Mb on the same chromosome or if the variant and the CpG were located on different chromosomes. Intrachromosomal *trans-*associations within 2 and 20 Mb for CpG that also shared a *cis-*meQTL were considered as long-range *cis-*meQTL and were excluded from the analysis.

#### Multiple testing correction for meQTL mapping

We applied permutation testing to determine significant thresholds for both *cis-* and *trans-*meQTLs. For each permutation, genotypes were randomly reassigned to bulls while maintaining the original EuroGMD SNP-based GRM. This approach accounted for population structure while disrupting the link between genotype and phenotype. To reduce the computational burden, GWASs were performed for 10% of CpGs (randomly selected) with permuted genotypes, and the lowest *P*-value (for *trans-*meQTLs), as well as the lowest *P*-value within 1 Mb (for *cis-*meQTL), were retained to approximate the distributions of *P*-values under the null hypothesis. This process was repeated 10 times, similar to previous studies [29, 30]. The resulted significance thresholds were *P* < 1.38 × 10^−4^ for *cis-*meQTLs and *P* < 1.25 × 10^−8^ for *trans-*meQTLs, which corresponded to an empirical false positive rate of 5%. Supplementary Table 2 shows the result of each of the 10 permutations.

#### Identification of spurious *trans-*meQTLs

Some *trans* associations showed a specific pattern in which several CpGs, clustered within a few hundred to a few thousand bp, were almost exclusively associated with variants clustered in a different genomic region, sometimes spanning tens of Mb. To investigate these associations, we retrieved the reference genome sequence corresponding to the CpGs cluster and the associated group of variants and aligned them to the publicly available Holstein-Friesian genome assembly (accession number = PRJNA668863) using BLASTN [31] available online [32]. If the sequence of a CpG cluster aligned to the genomic region containing its *trans-*associated variants, the CpG-SNP *trans-*pairs were considered false positives and discarded from further analyses. One notable case involved a CpG cluster spanning 1152 bp on BTA3 associated with variants located in a large region (40 Mb-65 Mb) on BTA9, for which BLASTN alignment of the CpG region did not identify a match in the BTA9 region defined by the variants with the significant effects. To further investigate this CpG cluster, we built a partial pangenome of the 35 Mb-70 Mb region on BTA9 using the reference genome and 8 Holstein genome assemblies (accession number = PRJEB68295). This was achieved through reference-free all-to-all alignment using PanGenome Graph Builder [33]. The resulting pangenome graph was visualized with BandageNG [34] to assess potential alignments and structural variations. This tool allowed BLAST searching of the CpG region within this pangenome.

#### Heritability

GRM-based heritability $\:\left({h}_{CpG}^{2}=\:\frac{{\sigma\:}_{u}^{2}}{{\sigma\:}_{u}^{2}+\:{\sigma\:}_{e}^{2}}\right)$ was estimated individually for each CpG. Estimates of variance explained by the EuroGMD variants ($\:{\sigma\:}_{u}^{2}$) and residual variance ($\:{\sigma\:}_{e}^{2}$) were obtained from the REML method implemented in GCTA [28].

### Functional annotations

CpGs and SNPs were annotated according to several datasets. First we derived annotations retrieved from Ensembl v112 [35]. Each CpG or variant was assigned to a genomic feature following this priority order: TSS200 (0-200 bases upstream of the transcription start site (TSS)) > TSS1500 (0-1500 bases upstream of the TSS) > 5′ UTR >1 st exon > 3′UTR > body > intergenic. We also retrieved CGI coordinates from the UCSC genome browser (“cpgIslandExt” table) [36]. To further characterize the CGI landscape, we have defined two additional regions around the CpG islands: (1) CGI shore regions, located 0-2 kb from CpG islands and (2) CGI shelf regions, located 2-4 kb regions from CpG islands. CpGs and SNPs outside these regions were assigned to the “open sea” feature. Genomic coordinates of Transposable Elements (TE) were obtained from the UCSC genome browser’s RepeatMasker Table [36], focusing on regions annotated as LTR, SINE, LINE (class I Retrotransposons), and DNA (class II Transposons). In addition to these annotations, we have also made use of a recent bovine TSS database obtained from Cap Analysis Gene Expression sequencing from the H2020 BovReg project [37], differentiating TSS reported in testis and ubiquitous TSS (minimum coverage of 10 reads and, commonly detected in a minimum of 66 out of the 102 tissues utilized in the study). For the TSS in testis, we annotated CpGs or SNPs using the following priority order: TSS+−200 (200 bp around the TSS) > TSS+−1500 (1500 bp around the TSS) > intergenic. For the ubiquitous TSS, the pattern was similar: TSS+−200 > TSS+−1500 > intergenic. Finally, we included ATAC-seq peak annotations from various cattle tissues (obtained from Yuan et al. [38]), retaining only peaks classified under the largest nonnegative matrix factorization components as “ubiquitous” or “testis”.

### Enrichment analyses

We computed odds ratios (OR) to look for enrichment and depletion in genomic regions. As neighbouring CpGs may be highly correlated and share a common methylation pattern, enrichment analyses were performed by considering a reduced set of “independent” CpGs. From the 166,985 CpGs, all adjacent CpGs with a correlation (R) > 0.5 were grouped into clusters and the genomic position considered was defined by its central CpG, resulting in 103,719 independent CpGs (see Supplementary Table 3 for the detailed list). We first tested enrichment of CpGs (*N* = 103,719) by genomic annotation to determine whether some genomic regions were enriched for CpGs with $\:{h}_{CpG}^{2}$>0.1 as described in Huan et al. [10], using 2-sided Fisher exact test. *P*-values were adjusted for multiple comparisons across two genomic annotation categories (CGI, genomic features). We then performed separate enrichment analysis for *cis-* and *trans-*CpG, along with their corresponding most significant *cis-* and *trans-*SNP (“top SNP”), considering only independent CpGs. For each, the OR was calculated against a randomly selected background set of the same size as the set of interest. This procedure was repeated 1,000 times to obtain the mean OR and 95% confidence interval (CI). For *cis-* and *trans-*CpGs, the background set consisted of randomly selected CpGs from all the independent CpGs (*N* = 103,719). For top *cis-*SNPs, we randomly selected the background set from all *cis-*SNPs tested (approximately 1.5 billion associations CpG-SNP) while maintaining MAF and distance to CpG similar to the one observed in the top *cis-*SNP set (set of interest). For that, we used four MAF classes ([0.03; 0.05[, [0.05; 0.1[, [0.1; 0.2[, [0.2; 0.5]) and seven distance classes relative to the corresponding CpG ([−1 Mb; −500 kb[, [−500 kb; −100 kb[, [−100 kb; −10 kb[, [−10 kb; 10 kb[, [10 kb; 100 kb[, [100 kb; 500 kb[, [500 kb; 1 Mb]). For the top *trans-*SNPs, the background set was randomly selected from the full 12.9 million variants set, while maintaining the MAF proportion (classes mentioned above) observed for the top *trans-*meQTL SNPs set.

### *Trans-*meQTL hotspots

We further investigated *trans-*meQTLs and their associated CpGs (*trans*-CpGs) by identifying *trans-*meQTL hotspots. For each lead SNP, defined as the SNP associated with the highest number of associated *trans-*CpGs (more than 30 as suggested by Huan et *al.* [10]*)*, we calculated LD with all the adjacent SNPs within a ± 2 Mb window around the lead SNP. SNPs with a high LD (r^2^ > 0.8) with the lead variant were retained, and these regions were defined as *trans-*meQTL hotspots. Next, we manually curated genes within or close to the previously defined hotspot intervals, focusing on genes involved in epigenetic processes. To facilitate this, we used an external database (Epifactors database [39]), which contains 802 genes involved in epigenetic processes, and checked for overlaps with the *trans-*meQTL hotspots.

### Enrichment of *trans-*meQTL hotspots CpG in transcription factor binding sites

The enrichment in transcription factor binding sites (TFBS) was examined for the CpGs of each *trans-*meQTL hotspot. The background set consisted of all the CpGs analyzed. 841 motifs corresponding to the JASPAR 2022 CORE vertebrates non-redundant release [40] were tested. We considered a 100-bp CpG window [9], centered on the CpG, and for CpGs that were less than 100 bp apart, the window was set from 50 bp before the first CpG to 50 bp after the last CpG. Following the merging of closely located CpGs, only the *trans-*meQTL hotspots with more than 20 sequences were retained. Enrichment were computed using SEA [41] and were considered significant when the false discovery rate (FDR) adjusted *P*-value (i.e., q-value) was below 0.05.

### Effect of sperm *trans-*meQTL hotspots in PBMCs

To investigate the effects of the *trans-*meQTL hotspots identified in sperm on PBMC DNAm, we examined the CpGs targeted by lead SNPs of the *trans-*meQTL hotspots in PBMCs. This analysis was conducted in a cohort of 54 bulls, which were also part of the sperm meQTL mapping design. For each *trans-*meQTL hotspot, we plotted the DNAm level of the associated *trans-*CpGs in PBMCs, classified by genotypes. If the observed DNAm pattern in PBMCs did not align with those observed in sperm, we concluded that the *trans-*meQTL hotspot was not shared between sperm and PBMCs for the investigated *trans*-CpGs.

## Results

### Selection of CpGs with variable DNA methylation

**Fig. 1:**
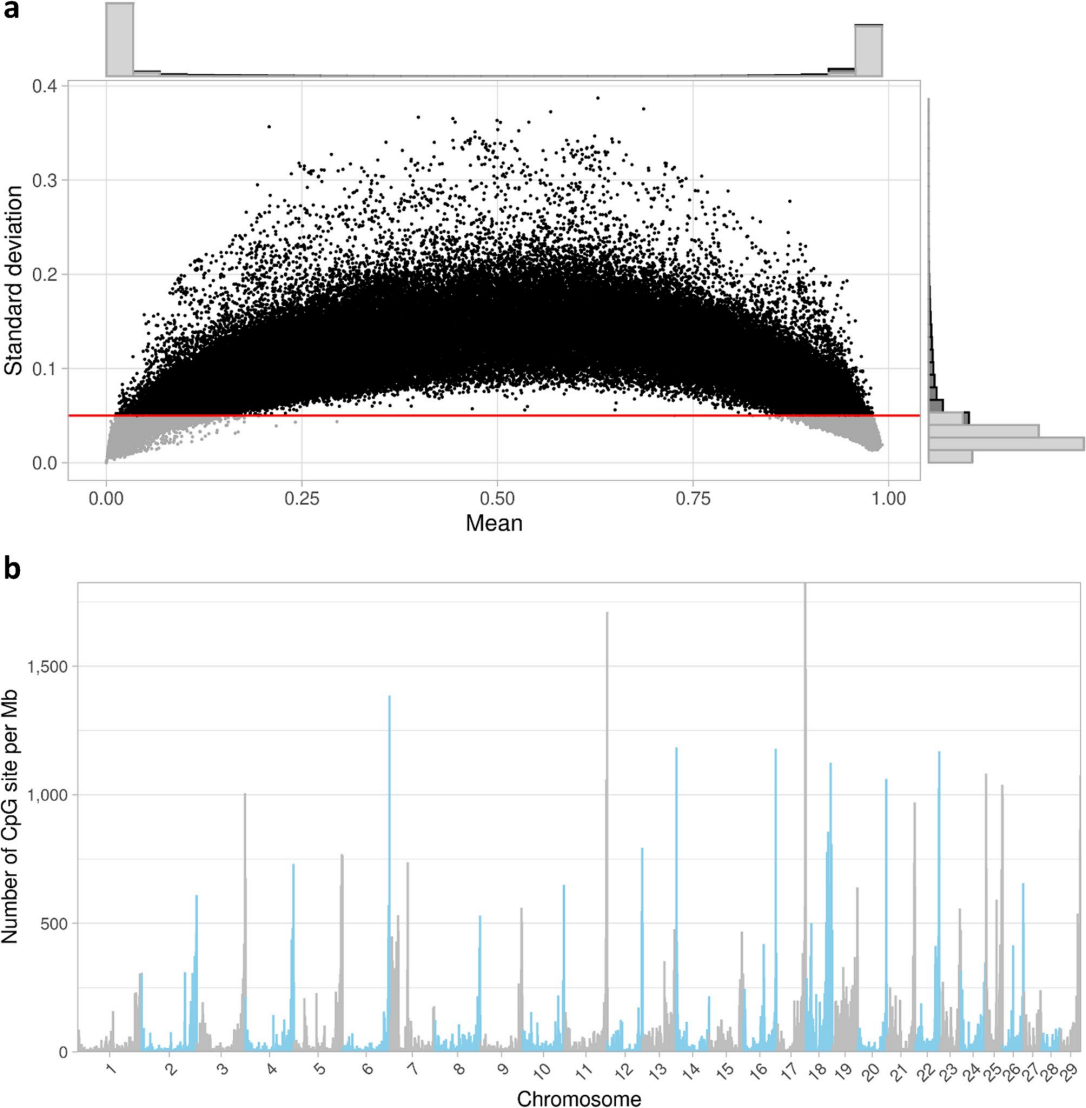
DNA methylation measured by RRBS. (**a**) Scatter plot of CpG methylation levels, showing the mean methylation ratio (x-axis) vs. the standard deviation (s.d.) (y-axis) for all analyzed CpGs (*N* = 1,212,361). Black dots, above the red line (s.d. >0.05), were retained for the study (*N* = 166,985). The upper and right-sided histograms represent the overall distribution of methylation levels and standard deviations, with retained CpGs highlighted in dark grey. (**b**) Distribution of the retained CpGs (*N* = 166,985) per Mb across the genome, with alternating colors distinguishing chromosomes

From the sperm samples of 405 marketed Holstein bulls, we identified and selected 166,985 CpGs non-overlapping with a known DNA polymorphism and with a DNAm s.d. above 5%. These selected CpGs represented 13.7% of the total number of CpGs covered by RRBS (*N* = 1,212,361) and excluded most CpGs with extreme DNA methylation values (Fig. 1a). The distribution of retained CpGs varied notably across autosomes, with counts ranging from 1,493 CpGs on BTA28 to 12,600 CpGs on BTA18 (Fig. 1b). These CpGs with variable DNAm were subsequently analyzed for genetic association using GWAS on imputed whole-genome sequences for Holstein bulls. The numbers and proportions of CpGs reported in the following results are therefore relative to the 166,985 CpGs with variable DNAm.

### Heritability of DNA methylation in sperm

**Fig. 2:**
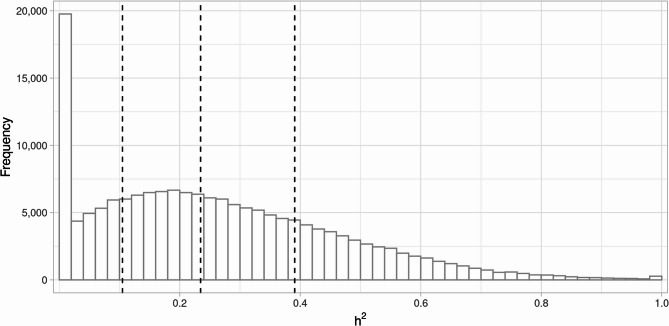
Distribution of heritability estimates ($\:{h}_{CpG}^{2}$) for 166,985 CpGs with variable DNA methylation. Dashed lines represent the first quartile, the median and the third quartile

Heritability of DNAm ($\:{h}_{CpG}^{2}$) was estimated for the 166,985 CpGs, and the distribution of heritability estimates is shown in Fig. 2 (see Supplementary Table 4 for individual values). The average $\:{h}_{CpG}^{2}$ across all CpGs was 0.26. Notably, 90% of CpGs had non-zero heritability estimate, and 76% had $\:{h}_{CpG}^{2}$ estimates > 0.1. Compared to the background set of 103,719 independent analyzed CpGs (bearing non redundant methylation information), independent CpGs with $\:{h}_{CpG}^{2}$ estimates > 0.1 were significantly enriched in intergenic regions (odds ratio (OR) = 1.21, *P* = 2.7 × 10^−33^) and in regions of low CpG density (OR = 1.36, *P* = 6.3 × 10^−107^), denoted “open-sea” regions, in contrast to CpG islands (CGI). Conversely, these CpGs were depleted in the 0–200 bp region upstream of transcription start sites (TSS200), gene bodies, CGI, CGI shores, and CGI shelves. Odds ratios and corresponding *P*-values are summarized in Table 1.

**Table 1:** Characterization of the CpGs with variable DNA methylation (*N* = 166,985) by genomic region: counts, mean heritability ($\:{h}_{CpG}^{2})$, and enrichment analysis results for the independent CpGs with $\:{h}_{CpG}^{2}$>0.1 compared to the 103,719 independent CpGs^a^

Genomic region		Number of variable CpGs	$\:{h}_{CpG}^{2}$ mean ±s.d.	Number of independent CpGs^a^	Enrichment for$\:{h}_{CpG}^{2})$ >0.1	
					Odds Ratio	*P*-value
Genomic features	TSS 200^b^	2726 (1.6%)	0.24 ± 0.19	1830 (1.8%)	0.83	1.3 × 10^−3^
	TSS 1500^c^	10,071 (6%)	0.25 ± 0.20	6844 (6.6%)	0.99	1
	5’UTR	2215 (1.3%)	0.26 ± 0.20	1327 (1.3%)	0.83	8.8 × 10^−3^
	1 st exon	5842 (3.5%)	0.27 ± 0.21	3330 (3.2%)	0.87	7.6 × 10^−4^
	Gene body	97,553 (58.4%)	0.26 ± 0.20	60,190 (58%)	0.91	5.1 × 10^−12^
	3’UTR	6019 (3.6%)	0.24 ± 0.18	3972 (3.8%)	0.99	1
	Intergenic	42,559 (25.5%)	0.28 ± 0.20	26,226 (25.3%)	1.21	2.7 × 10^−33^
CpG landscape^d^	CGI	60,466 (36.2%)	0.26 ± 0.20	33,756 (32.5%)	0.8	4.1 × 10^−54^
	CGI shores	41,433 (24.8%)	0.25 ± 0.19	28,232 (27.2%)	0.94	2.1 × 10^−4^
	CGI shelves	9854 (5.9%)	0.24 ± 0.20	6571 (6.3%)	0.87	2.2 × 10^−6^
	open sea	55,232 (33.1%)	0.28 ± 0.21	35,150 (33.9%)	1.36	6.3 × 10^−107^

^a^Adjacents CpGs with DNAm R >0.5 were grouped

^b^TSS 200: 0–200 bases upstream to transcription start sites (TSS)

^c^TSS 1500: 0–1500 bases upstream to TSS

^d^CGI: CpG island; CGI shores: 0-2kb from CGI; CGI shelves: 2-4kb regions from CGI; open-sea: >4kb regions from CGI

### Methylation QTL mapping

**Fig. 3:**
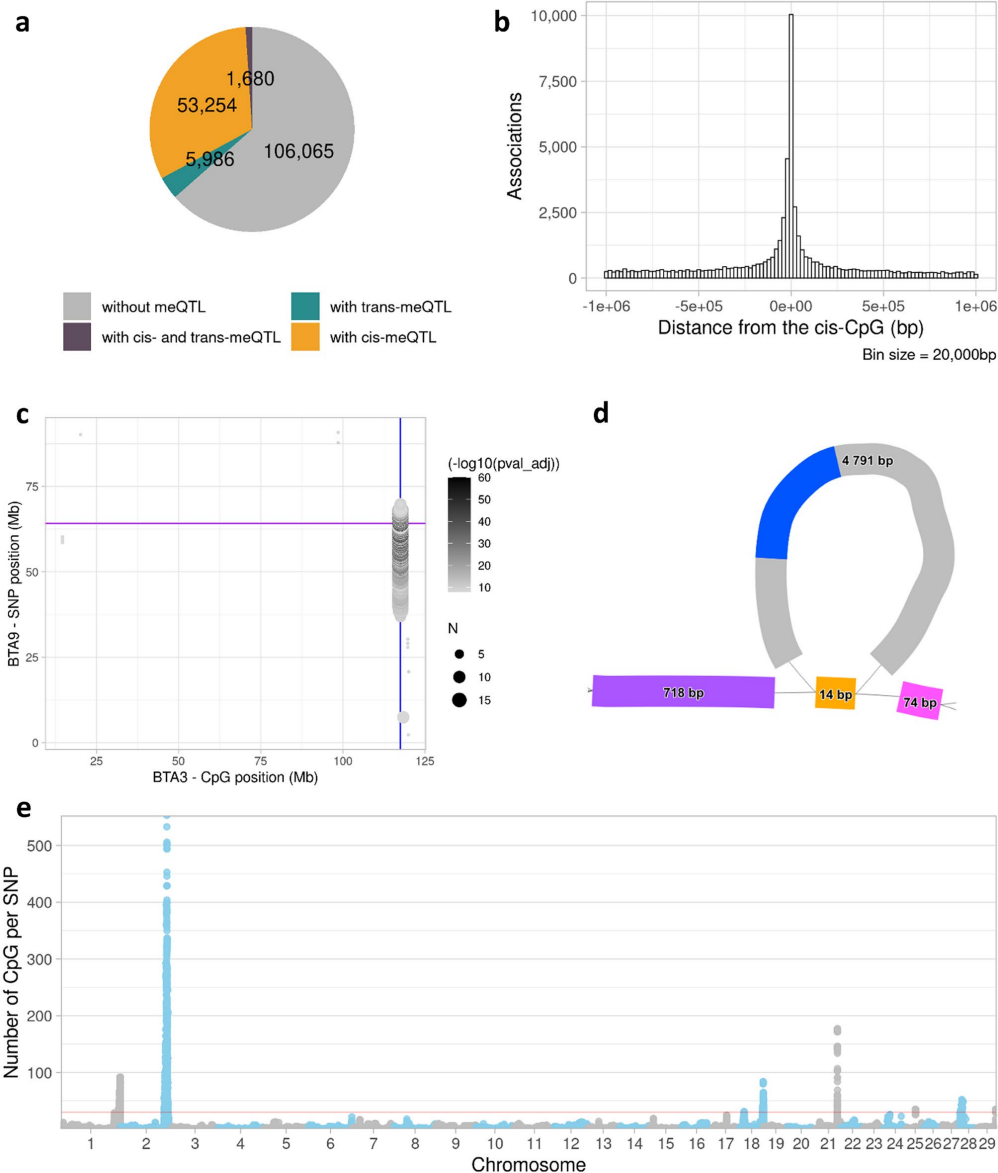
Mapping of *cis-* and *trans-*methylation QTLs. (**a**) Distribution of meQTLs types for the 166,985 CpGs. (**b**) Distribution of the distance between the CpG and the top *cis-*SNP. (**c**) Significant *trans* associations between SNPs located on BTA9 and CpGs located on BTA3. “*N*” indicates the number of associated *trans-*CpG per SNP. Purple line = BTA9:64,209,331 − 64,210,048 bp. Blue line = BTA3:117,543,159 − 117,544,310 bp (containing up to 19 *trans-*CpGs). (**d**) Pangenome bubble within the 35 to 70 Mb region of BTA9 extracted from a pangenome graph constructed with ARS-UCD1.2 and 8 Holstein genome assemblies. Purple segment = BTA9:64,209,331 − 64,210,048 bp. Pink segment = BTA9:64,209,243 − 64,209,316 bp. Blue segment = region with a 99.74% identity with BTA3:117,543,159 − 117,544,310 bp. The reported genomic coordinates corresponded to positions within the ARS-UCD1.2 reference genome. (**e**) Number of *trans-*CpGs (y-axis) associated to each SNP (chromosome coordinates on x-axis). Red line = 30 CpGs

GWAS were performed for all 166,985 CpGs to identify both *cis-* (within 1 Mb upstream or downstream of the associated CpG) and *trans-*meQTLs (intrachromosomal > 2 Mb away or interchromosomal from the associated CpG). Intrachromosomal associations between 1 and 2 Mb were considered potential long-range *cis* associations and were excluded. At a significance threshold of *P* < 1.38 × 10^−4^ (Supplementary Fig. 1), a total of 57,450,466 significant *cis-*associations were detected, representing *cis-*meQTLs for 54,934 CpGs (32.9% of the analysed CpGs) (Fig. 3a). On average, a *cis-*CpG was located 260.8 kb (absolute mean; s.d. = 299.4 kb; median = 114.2 kb) away from its top *cis-*SNP (defined as the associated SNP with the lowest *p*-value). The median number of SNPs per *cis-*CpG was 384 (mean = 1,046, s.d. = 1,434). Figure 3b shows the distribution of distances between CpGs and their top *cis-*SNPs. The top *cis-*SNPs and associated *P*-values are reported in Supplementary Table 4.

At a threshold of *P* < 1.25 × 10^−8^ (Supplementary Fig. 1), we identified 17,593,537 *trans-*CpG-SNP pairs involving 23,327 CpGs. These associations included intrachromosomal pairs (> 2 Mb apart) and interchromosomal pairs. A large proportion of *trans* associations (79.3%) involved intrachromosomal CpG-SNP pairs with a genomic distance within 2 Mb to 20 Mb. Among these 2–20 Mb intrachromosomal associations, 99.4% of the associated CpGs also had a *cis-*meQTL. Since these associations are likely to result from long-range LD, they were excluded from further *trans-*meQTL analyses. Furthermore, to address potential mapping inaccuracies and structural polymorphisms, we aligned several sequences defined by groups of CpGs or SNPs to a Holstein-Friesian genome assembly (accession number = PRJNA668863) different from the Hereford reference genome (ARS-UCD1.2). This verification step was conducted on genomic regions for which SNPs were associated with at least 10 distant CpGs within the same genomic region. This revealed 11 regions (ranging from 54 to 196,313 bp) that aligned near their respective *trans-*SNPs or *trans-*CpGs, with > 91% query coverage and > 98.48% of identity (Supplementary Table 5). These associations were discarded as they were likely false positive *trans-*associations. For one region, showing a strong association between SNPs located on BTA9 and a cluster of 19 CpGs spanning over 1,152 bp on BTA3 (Fig. 3c), the alignment with the Holstein-Friesian genome assembly did not reveal mapping errors. However, the presence of such an exclusive long-range association between these CpGs and distant SNPs is biologically intriguing. To investigate further this region, we constructed a reduced pangenome graph using the reference genome and eight Holstein *de novo* genome assemblies (Fig. 3d). We identified a 4,791 bp structural variant (SV) on BTA9 corresponding to an insertion (carried by one Holstein individual) relative to the reference genome located between 64,209,316 and 64,209,331 bp (ARS-UCD1.2 coordinates). This SV contained, with 99.74% identity, the 1,152 bp segment initially located on BTA3 on the reference genome. Therefore, associations involving this structural variant were excluded from the *trans-*meQTL analyses. After applying all filters, 2,422,352 *trans-*CpG-SNP pairs, corresponding to 7,666 CpGs (4.6% of all CpGs) remained (top *trans-*SNPs are reported in Supplementary Table 4). Of these, 440 CpGs had intrachromosomal *trans-*meQTL, and 1,680 CpGs (1.0%) had both *cis-* and *trans-*meQTLs (Fig. 3a). In total, 680,122 SNPs were associated with at least one *trans-*CpG, with 1.2% (8,082 SNPs) linked to 30 or more *trans-*CpGs, as shown in Fig. 3e.

### Genomic enrichments of meQTLs and their CpGs

**Fig. 4:**
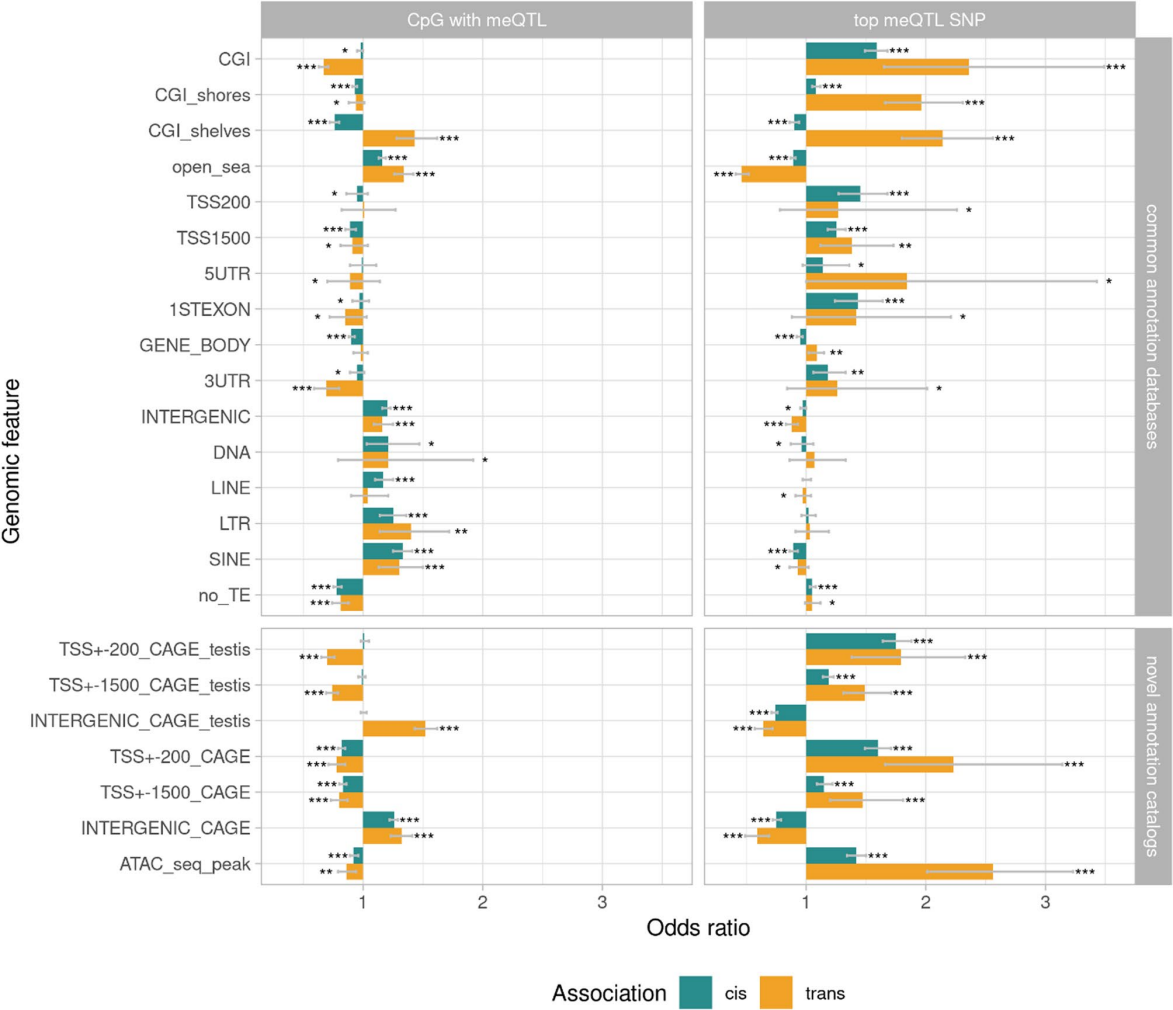
Enrichment in genomic annotations of independent *cis-*CpGs, *trans-*CpGs and their corresponding top *cis-* and *trans-*meQTL SNPs (selected by lowest *P*-value) for common annotation databases (“cpgIslandExt” Table [36] from “CGI” to “open_sea”; Ensembl release 112 [35] from “TSS200” to “INTERGENIC”; and RepeatMasker [36] from “DNA” to “SINE”) or novel catalogs (CAGE annotation track [37] from “TSS+−200_CAGE_testis” to “INTERGENIC_CAGE” and ATAC-seq bovine catalog [38] from “ATAC_seq_peaks”, from which only “ubiquitous” or “testis” peaks were retained). The error bars represent the 95% confidence interval. *P < 0.05, **P < 0.01, ***P < 0.001

We performed enrichment analyses distinguishing independent *cis-*CpGs, *trans-*CpGs and their corresponding top *cis-*SNPs and top *trans-*SNPs (based on the lowest *P*-value). OR and 95% confidence interval are shown in Fig. 4 and reported in Supplementary Table 6. Overall, *cis-* and *trans-*CpGs showed moderate enrichment patterns toward functional elements (0.67 < OR < 1.57). In contrast, the corresponding top SNPs presented stronger over- and under-representation patterns. With regards to the CpG Island (CGI) landscape, both CGI (OR_cis_ = 1.59, OR_trans_ = 2.36) and CGI shores (OR_cis_ = 1.08; OR_trans_ = 1.96), were significantly enriched for top *cis-* and *trans-*meQTL SNPs, while open-sea regions were depleted (OR_cis_ = 0.89; OR_trans_ = 0.46). Both top *cis-* and *trans-*meQTL SNPs were overrepresented in TSS200, TSS1500, and 3’UTR regions (OR > 1) and the *trans-*meQTL SNPs were under-represented in intergenic regions (OR < 1). Furthermore, the meQTL SNPs were preferentially located in regions depleted of transposable elements (“no_TE”: OR > 1), while their associated CpGs showed enrichment for TEs (depletion in “no_TE”: OR_cis_ = 0.78; OR_trans_ = 0.81), in particular Long Terminal Repeats (LTR: OR_cis_ = 1.25; OR_trans_ = 1.40) and Short Interspersed Repetitive Elements (SINE: OR_cis_ = 1.33; OR_trans_ = 1.30) SINE. When analyzing TSS surrounding regions annotations (TSS ± 200 and TSS ± 1500) derived from TSS recently identified by Cap Analysis Gene Expression sequencing (CAGE) [37], we found consistent meQTL SNPs enrichment for both testis-specific TSS (“CAGE_testis”) and ubiquitous TSS (“CAGE”). For the TSS ± 200 regions, this enrichment was stronger compared to Ensembl derived TSS ± 200 annotation [35] (TSS ± 200 in testis: OR_cis_ = 1.75; OR_trans_ = 1.79; ubiquitous TSS ± 200: OR_cis_ = 1.60; OR_trans_ = 2.23). Lastly, the top meQTL SNPs were enriched in combined testis-specific and ubiquitous ATAC-seq peaks (regions of open chromatin) (OR_cis_ = 1.42; OR_trans_ = 2.56), in line with the observed enrichment in TSS regions. This enrichment was particularly marked for *trans-*meQTLs.

### Investigation of* trans-*meQTL hotspots

*Trans-*meQTL mapping revealed the presence of several genomic regions associated with multiple *trans-*CpGs. We focused on *trans-*meQTL hotspots, defined as regions associated with more than 30 *trans-*CpGs (Table 2). A total of eight *trans-*meQTL hotspots (H1 to H8) were identified on seven autosomes, collectively targeting 1,058 *trans-*CpGs, representing 481 independent *trans-*CpGs (considered by a DNAm *r*^2^ < 0.25 between two adjacent *trans-*CpGs). For each *trans*-meQTL hotspot, genomic intervals were defined using all variants in high LD (i.e., *r*^2^ > 0.8) with the lead variant (associated with the highest number of *trans-*CpGs) located within ± 2 Mb. For each interval, we manually curated candidate genes in the hotspot regions, retaining only genes known to be involved in epigenetic regulation.

**Table 2:** Description of eight *trans-*meQTL hotspots

hotspot	BTA	Interval (in Mb)		Most significant SNP				Candidate genes
			Position	#* trans*-CpGs		*trans*-CpGs with opposite effects to the hotspot majority (%)	# independent CpGs^a^	
H1	1	156.8-157.6	157,443,428	92		10.9	76	*KAT2B*^*b*^, *PRDM9*^*b*^
H2	2	120.5–123.0	122,297,963	553		9.2	214	*RBBP4*^*b*^, *HDAC1*^*b*^, *SPOCD1*
H3	18	12.7–13.9	13,370,307	31		0	10	*BANP*^*b*^
H4	18	63.8–63.9	63,854,493	84		8.3	31	*PRPF31*^*bc*^, *TFPT*^*bc*^
H5	21	60.3–60.5	60,522,514	177		0	91	*TCL1B*,* TCL1A*^*bc*^
H6	25	21.9–22.3	22,201,331	35		0	15	*PRKCB*^*bc*^
H7	28	4.6–6.5	5,294,954	51		0	24	*-*
H8	29	48.7–48.8	48,763,272	35		0	20	*NAP1L4*^*bc*^

^a^Adjacents CpGs with DNAm R >0.5 were grouped

^b^In Epifactor database [39]

^c^Outside the hotspot interval (distance from the interval: *PRPF31* = 560 kb, *TFPT* = 551 kb, *TCL1A* = 0.6 kb, *PRKCB* = 68 kb, *NAP1L4* = 59 kb)

The first *trans-*meQTL hotspot (H1) overlapped with several genes, including *KAT2B* (a histone acetyltransferase gene, HAT) and *PRDM9* (a histone methyltransferase, HMT), which catalyzes H3K4 trimethylation and has been reported to bind to recombination hotspots [42]. The lead variant of H2 (BTA2:122297963 C > T) was associated with 553 CpGs located across the 29 autosomes, with the T > C allele effect increasing DNAm by on average of 5.5% (s.d. = 4.6%), positive for 91% of the 553 CpGs. The interval of the second hotspot (~ 2.5 Mb) contained genes *HDAC1*, encoding a histone deacetylase enzyme (HDAC), a histone chaperone (*RBBP4*), and *SPOCD1*, a gene involved in piRNA directed *de novo* methylation of TE in the germline [43], which interacts with *DNMT3A* (DNA (cytosine-5)-methyltransferase 3 A) encoding an enzyme that catalyzes the *de novo* transfer of methyl groups to DNA. Of the two *trans-*meQTL hotspots contained in BTA18, the first one (H3) included *BANP*, a protein-coding gene that binds to matrix attachment regions [44] involved in chromatin structure and activates CGI [45]. *BANP* interacts with histone modifying enzymes, including *HDAC6* [46] and *HDAC1* [47]. No known gene overlaps the 22.7-kb interval of H4 on BTA18. At a distance of ± 0.6 Mb, two genes, *PRPF31* and *TFPT*, were found to be both components of complexes involved in epigenetic processes [39]. H5 overlapped with 4 genes, including *TCL1B*, the closest gene to the lead SNP. While *TCL1B* does not interact directly with *DNMT3A*, *TCL1A*, a highly similar gene located 26 kb from the lead variant, physically interacts with *DNMT3A* and *DNMT3B*, with *TCL1A* overexpression inhibiting *DNMT3A* [48] activity. The H6 lead variant was located 320 kb away from *PRKCB*, a histone kinase gene. Although several genes overlapped with the H7 interval, we did not identify any epigenetic regulatory genes. *NAP1L4*, a histone chaperone gene, is located close to the H8 hotspot. Of the eight hotspots, five (H3, H5, H6, H7, and H8) had unidirectional estimated effects on their associated *trans-*CpGs. H1 showed the highest frequency (11%) of *trans-*CpGs with effects opposite to the majority. In comparison with the full set of analyzed CpGs (*N* = 166,985), no significant enrichment in transcription factor binding sites (TFBSs) was observed for CpGs associated with any *trans-*meQTL hotspots (5% FDR adjusted *P*-value > 0.05).

We then investigated whether the lead variants from the *trans*-meQTL hotspots affecting DNAm at more than 30 *trans-*CpGs in sperm also affected DNAm at the same *trans-*CpGs in peripheral blood mononuclear cells (PBMCs). PBMC DNAm data, also measured with RRBS, was available for 54 of the 405 bulls. Hotspots H7 and H8 were not considered in this analysis due to the low number of *trans-*CpGs shared between sperm and PBMCs (5 CpGs, 9.6%; and 7 CpGs, 20%, respectively). The remaining hotspots shared DNAm data for at least 78.2% of their *trans-*CpGs between the two tissues. To assess genetic influence, we plotted the DNAm levels of the *trans-*CpGs across sperm and PBMCs for each hotspot, stratified by genotypes of the lead variant. The results showed no evidence of a genetic influence of the lead variants on DNAm levels of the shared *trans-*CpGs in PBMCs (Supplementary Fig. 2).

## Discussion

We performed meQTL mapping analyses for 166,985 CpGs with variable DNAm and 12,930,389 variants to investigate the genetic architecture of sperm DNAm in bulls. Paving the way for further studies aimed at integrating epigenetic information into the prediction of performance traits (e.g., milk yield, fertility), we reported that, on average, genetic variance accounted for 26% of the DNAm variability, with heterogeneity observed across different genomic features. We identified *cis-*meQTLs for approximately one-third of the CpGs analyzed and *trans-*meQTLs for 4.6% of CpGs. These proportions are consistent with previous studies [5, 6, 49, 50], despite significant differences in experimental design, including: (1) the tissue analyzed (sperm vs. blood), (2) the species studied (cattle vs. human), (3) the technology used for methylation measurement (RRBS vs. DNAm array), and (4) the germline-specific epigenetic reprogramming. Additionally, we found eight *trans-*meQTL hotspots, of which four overlapped with genes involved in epigenetic processes. Interestingly, the genetic effects on associated CpGs in PBMCs did not mirror the patterns observed in sperm. Further research is needed to determine whether this observation reflects a sperm-specific mechanism involved in the genetic regulation of DNAm.

Highly variable CpGs have been reported to be more heritable than less variable ones [6], which is an important consideration in our study, as we selected CpGs based on their individual variability. This observation may be attributed to the technical variability, which is proportionally larger when total variability is low. Besides, the majority of the CpGs have non-variable DNAm and we can hypothesize that they are under strong genetic control. Despite the selection criterion based on variability, our heritability estimates were similar to those derived from DNAm array data in human blood, even though they were estimated in a different tissue [5], corroborating our findings. Heritable CpGs ($\:{h}_{CpG}^{2}$ >0.1) are depleted in TSS200 regions and in high-CpG dense regions, as also observed in human blood data [10].

Without considering transposable elements, the general enrichment of meQTLs in specific genomic annotations (e.g., CGI, TSS surrounding regions) and the depletion of open-sea and intergenic regions were similar to observations reported by Villicaña et al. [6]. These findings remain consistent despite comparing results across different tissues and species. Min et al. [8] underpinned that variants associated with meQTLs are enriched in chromatin states, although the enrichments depended on the type of association (active chromatin domains for *cis* and *cis-* + *trans-*meQTLs, heterochromatin for *trans-*meQTLs). Using recently published bovine ATAC-seq data [38], we observed significant over-representation of *cis-* and *trans-*meQTLs in ATAC-seq peaks, which mark regions of open chromatin, corroborating that regulatory variant preferentially map to tissue-specific ATAC-seq peaks (as described in Yuan et al. [38]). Enrichment was also observed in TSS ± 200 regions derived from a recent and improved TSS annotation track based on CAGE-seq data [37], with notably higher enrichment levels compared to TSS200 regions annotations from Ensembl v112 [35].

While the DNAm landscape is highly tissue-specific and dependent on the developmental stage [51], several studies have identified shared meQTLs across tissues, such as blood, various brain regions [52, 53], and saliva [53], although these associations were examined over short genomic distances. For example, Lin et al. [53] reported 45–73% overlap in *cis-*meQTL among tissues. To our knowledge, cross-tissue meQTLs comparisons have not yet included a direct blood-sperm comparison analysis. In the present study, we did not identify a shared genetic architecture for *trans-*meQTLs affecting multiple CpGs across both sperm and blood. However, because our analysis focused on genetic variants affecting multiple distant CpGs, broader conclusions regarding the genetic regulation of DNA methylation across tissues should be drawn with caution. Following these observations, despite most meQTL studies focusing on blood, identifying sperm meQTLs is essential for addressing key scientific questions, including understanding the transmission of paternally inherited DNA marks [54].

Previous research has suggested that changes in DNAm may result from altered chromatin conformation [55]. Several studies investigating the genetic architecture of DNA methylation have identified histone modifier genes [55, 56], highlighting the well-established interdependence between histones and DNA methylation [57]. In this study, the reported candidate genes overlapping the *trans-*meQTL hotspots align with this hypothesis. Specifically, chromatin in male germ cells exhibits a unique structure, characterized by extreme compaction, with most histones substituted by protamines [58]. Notably, two of the identified candidate genes from *trans-*meQTL hotspots investigations, *PRDM9* and *SPOCD1*, are particularly compelling due to their active roles during spermatogenesis. *PRMD9* acts as a hotspot regulator through a *trans-*acting mechanism [59], aligning with our findings. *SPOCD1* is involved in piRNA-mediated DNA methylation targeting TE and its deficiency leads to male-specific infertility [43]. This is aligns with a tendency for LINE L1 hypomethylation in subfertile bulls [24], thus reinforcing the interest in understanding the underlying mechanisms.

Exploration of *trans-*meQTL mapping revealed several spurious *trans-*associations between CpGs and variants, likely arising from inaccuracies in the reference genome for our population. These inaccuracies were confirmed by analyses of multiple genome assemblies. More broadly, the search for associations between genomic markers located in different genomic regions, i.e., SNPs and CpGs, underlined a general pattern: a high proportion of short-range associations but very few long-range ones. Strong and exclusive associations between distant genomic regions are rare and may result from assembly errors or structural variants. The construction of a cattle pangenome has facilitated the discovery of novel structural variants (SVs) molecular QTLs [60] (molQTLs; i.e., to refer to molecular phenotypes such as gene expression data, protein abundance, and methylation). Here, we extended this concept, emphasizing that pangenomes can also refine molQTL mapping by highlighting SVs, which allow the detection and elimination of spurious distant associations. However, the pangenome approach is computationally demanding and requires a large and diverse set of genomes to increase the probability of observing rare events.

The extensive catalog of sperm meQTLs generated in this study represents a valuable resource to improve our understanding of the biological mechanisms underlying complex traits. In cattle, DNA methylation has already been associated with traits of economic importance [61, 62]. Specifically regarding field fertility, Costes et al. [24] identified distinct methylation patterns between fertile and sub-fertile Montbéliard bulls. Building on these findings, integrating meQTL data with GWAS results and fertility-associated methylation signatures offers a promising avenue to uncover the molecular mechanisms involved in bull fertility. SNPs that influence DNA methylation in sperm may contribute to variation in gene regulation or TE activity during spermatogenesis or early embryonic development, potentially affecting fertility outcomes. By identifying loci where genetic variants modulate epigenetic marks, we can begin to disentangle causal pathways and prioritize candidate genes or regulatory elements for functional validation. Ultimately, this integrative approach could improve the accuracy of fertility-related trait prediction and inform breeding strategies.

While our findings are promising, several limitations of our study should be acknowledged. First, the CpGs analyzed were selected from a population of CpGs generated by RRBS (using the MspI restriction enzyme), a technology that does not provide a uniform representation of CpGs across the genome [63] (bias towards CpG-dense regions). In addition, the CpGs were selected solely on the basis of their individual variability. As a result, the analyzed CpGs were not entirely independent, as physically proximal CpGs may show similar DNAm patterns. The use of independent CpGs in enrichment analysis attempted to overcome this phenomenon. Third, the limited sample size may have increased the risk of false positives or overestimation of effect size [64]. Although we controlled for false positive rate using permutations, the limited sample size reduced our ability to identify rare variants affecting DNA methylation. The use of widely available epigenotyping tools, such as DNAm chips, could enable larger-scale phenotyping efforts, although outcomes would depend on marker selection and the tissue studied.

## Conclusions

The identification of molQTLs is the subject of intense research to gain a better understanding of the links between genes and phenotypes. To this end, we mapped both *cis-* and *trans-*meQTLs in bull semen. In particular, we showed that the identification of *trans-*meQTLs is sensitive to the reference genome assembly, which can lead to spurious associations. Our results also support the hypothesis that the influence of genetic variants on multiple CpGs may be mediated through changes in chromatin structure during germ cell differentiation, possibly involving histone modifiers genes. These findings further our understanding of the genetic architecture of bovine semen methylation, a relatively unexplored field.

## Supplementary Information

Supplementary Material 1.

Supplementary Material 2.

## Data Availability

RRBS fastq files are available through the NCBI SRA under accession number PRJNA1225939 (http://www.ncbi.nlm.nih.gov/bioproject/1225939). The fasta files of the eight 8 Holstein used to build the partial pangenome are available in the European Nucleotide Archive (ENA) at EMBL-EBI under accession number PRJEB68295 (https://www.ebi.ac.uk/ena/data/view/PRJEB68295). Genotypes of the 405 bulls are commercial information belonging to the French breeders and are not publicly available. All other datasets supporting findings are included in the article and its supplementary tables and figures.

## References

[1] Singal R, Ginder GD. DNA Methylation. Blood. 1999;93:4059–70. 10361102

[2] Moore LD, Le T, Fan G. DNA methylation and its basic function. Neuropsychopharmacol. 2013;38:23–38. 10.1038/npp.2012.112 PMC3521964 22781841

[3] Perino M, Veenstra GJC. Chromatin control of developmental dynamics and plasticity. Dev Cell. 2016;38:610–20. 27676434 10.1016/j.devcel.2016.08.004

[4] Leenen FAD, Muller CP, Turner JD. DNA methylation: conducting the orchestra from exposure to phenotype? Clin Epigenet. 2016;8: 92. 10.1186/s13148-016-0256-8 PMC5012062 27602172

[5] Villicaña S, Bell JT. Genetic impacts on DNA methylation: research findings and future perspectives. Genome Biol. 2021;22: 127. 33931130 10.1186/s13059-021-02347-6 PMC8086086

[6] Villicaña S, Castillo-Fernandez J, Hannon E, Christiansen C, Tsai P-C, Maddock J, et al. Genetic impacts on DNA methylation help elucidate regulatory genomic processes. Genome Biol. 2023;24: 176. 37525248 10.1186/s13059-023-03011-x PMC10391992

[7] McRae AF, Marioni RE, Shah S, Yang J, Powell JE, Harris SE, et al. Identification of 55,000 replicated DNA methylation QTL. Sci Rep. 2018;8:17605. 30514905 10.1038/s41598-018-35871-w PMC6279736

[8] Min JL, Hemani G, Hannon E, Dekkers KF, Castillo-Fernandez J, Luijk R, et al. Genomic and phenotypic insights from an atlas of genetic effects on DNA methylation. Nat Genet. 2021;53:1311–21. 34493871 10.1038/s41588-021-00923-x PMC7612069

[9] Hawe JS, Wilson R, Schmid KT, Zhou L, Lakshmanan LN, Lehne BC, et al. Genetic variation influencing DNA methylation provides insights into molecular mechanisms regulating genomic function. Nat Genet. 2022;54:18–29. 34980917 10.1038/s41588-021-00969-x PMC7617265

[10] Huan T, Joehanes R, Song C, Peng F, Guo Y, Mendelson M, et al. Genome-wide identification of DNA methylation QTLs in whole blood highlights pathways for cardiovascular disease. Nat Commun. 2019;10: 4267. 31537805 10.1038/s41467-019-12228-z PMC6753136

[11] Perzel Mandell KA, Eagles NJ, Wilton R, Price AJ, Semick SA, Collado-Torres L, et al. Genome-wide sequencing-based identification of methylation quantitative trait loci and their role in schizophrenia risk. Nat Commun. 2021;12: 5251. 34475392 10.1038/s41467-021-25517-3 PMC8413445

[12] Zhang T, Choi J, Dilshat R, Einarsdóttir BÓ, Kovacs MA, Xu M, et al. Cell-type-specific MeQTLs extend melanoma GWAS annotation beyond eQTLs and inform melanocyte gene-regulatory mechanisms. Am J Hum Genet. 2021;108:1631–46. 34293285 10.1016/j.ajhg.2021.06.018 PMC8456160

[13] Hazard D, Plisson-Petit F, Moreno-Romieux C, Fabre S, Drouilhet L. Genetic determinism exists for the global DNA methylation rate in sheep. Front Genet. 2020;11: 616960. 33424937 10.3389/fgene.2020.616960 PMC7786236

[14] Shen Q, Wang K, Wang S, Zhao Z, Ji X, Chen D, et al. Identifying key DNA methylation sites and their cis-methylation quantitative loci for intramuscular fatty acid traits using genome and methylome data in Yorkshire pigs. Italian J Anim Sci. 2023;22:829–40.

[15] Ribeiro AMF, Sanglard LP, Wijesena HR, Ciobanu DC, Horvath S, Spangler ML. DNA methylation profile in beef cattle is influenced by additive genetics and age. Sci Rep. 2022;12(1): 12016. 35835812 10.1038/s41598-022-16350-9 PMC9283455

[16] Smallwood SA, Kelsey G. De novo DNA methylation: a germ cell perspective. Trends Genet. 2012;28:33–42. 22019337 10.1016/j.tig.2011.09.004

[17] Zhou Y, Connor EE, Bickhart DM, Li C, Baldwin RL, Schroeder SG, et al. Comparative whole genome DNA methylation profiling of cattle sperm and somatic tissues reveals striking hypomethylated patterns in sperm. Gigascience. 2018;7:giy039. 29635292 10.1093/gigascience/giy039 PMC5928411

[18] Perrier J-P, Sellem E, Prézelin A, Gasselin M, Jouneau L, Piumi F, et al. A multi-scale analysis of bull sperm methylome revealed both species peculiarities and conserved tissue-specific features. BMC Genomics. 2018;19: 404. 29843609 10.1186/s12864-018-4764-0 PMC5975405

[19] Sanchez M-P, Guatteo R, Davergne A, Saout J, Grohs C, Deloche M-C, et al. Identification of the ABCC4, IER3, and CBFA2T2 candidate genes for resistance to paratuberculosis from sequence-based GWAS in Holstein and Normande dairy cattle. Genet Selection Evol. 2020;52:14. 10.1186/s12711-020-00535-9 PMC7077142 32183688

[20] Sargolzaei M, Chesnais JP, Schenkel FS. A new approach for efficient genotype imputation using information from relatives. BMC Genomics. 2014;15:478. 24935670 10.1186/1471-2164-15-478 PMC4076979

[21] Howie B, Fuchsberger C, Stephens M, Marchini J, Abecasis GR. Fast and accurate genotype imputation in genome-wide association studies through pre-phasing. Nat Genet. 2012;44:955–9. 22820512 10.1038/ng.2354 PMC3696580

[22] Sanchez M-P, Ramayo-Caldas Y, Wolf V, Laithier C, El Jabri M, Michenet A, et al. Sequence-based GWAS, network and pathway analyses reveal genes co-associated with milk cheese-making properties and milk composition in Montbéliarde cows. Genet Selection Evol. 2019;51:34. 10.1186/s12711-019-0473-7 PMC6604208 31262251

[23] Sahana G, Cai Z, Sanchez MP, Bouwman AC, Boichard D. Invited review: good practices in genome-wide association studies to identify candidate sequence variants in dairy cattle. J Dairy Sci. 2023;106:5218–41. 37349208 10.3168/jds.2022-22694

[24] Costes V, Chaulot-Talmon A, Sellem E, Perrier J-P, Aubert-Frambourg A, Jouneau L, et al. Predicting male fertility from the sperm methylome: application to 120 bulls with hundreds of artificial insemination records. Clin Epigenetics. 2022;14:54. 35477426 10.1186/s13148-022-01275-x PMC9047354

[25] Krueger F. Trim Galore! A wrapper around Cutadapt and FastQC to consistently apply adapter and quality trimming to FastQ files, with extra functionality for RRBS data. Babraham Institute. 2015. https://github.com/FelixKrueger/TrimGalore.

[26] Krueger F, Andrews SR. Bismark: a flexible aligner and methylation caller for Bisulfite-Seq applications. Bioinformatics. 2011;27:1571–2. 21493656 10.1093/bioinformatics/btr167 PMC3102221

[27] Daetwyler HD, Capitan A, Pausch H, Stothard P, Van Binsbergen R, Brøndum RF, et al. Whole-genome sequencing of 234 bulls facilitates mapping of monogenic and complex traits in cattle. Nat Genet. 2014;46:858–65. 25017103 10.1038/ng.3034

[28] Yang J, Lee SH, Goddard ME, Visscher PM. GCTA: A tool for Genome-wide complex trait analysis. Am J Hum Genet. 2011;88:76–82. 21167468 10.1016/j.ajhg.2010.11.011 PMC3014363

[29] Bonder MJ, Luijk R, Zhernakova DV, Moed M, Deelen P, Vermaat M, et al. Disease variants alter transcription factor levels and methylation of their binding sites. Nat Genet. 2017;49:131–8. 27918535 10.1038/ng.3721

[30] Morrow JD, Glass K, Cho MH, Hersh CP, Pinto-Plata V, Celli B, et al. Human lung DNA methylation quantitative trait loci colocalize with chronic obstructive pulmonary disease genome-wide association loci. Am J Respir Crit Care Med. 2018;197:1275–84. 29313708 10.1164/rccm.201707-1434OC PMC5955059

[31] Zhang Z, Schwartz S, Wagner L, Miller W. A greedy algorithm for aligning DNA sequences. J Comput Biol. 2000;7:203–14. 10890397 10.1089/10665270050081478

[32] Nucleotide BLAST. Search nucleotide databases using a nucleotide query. https://blast.ncbi.nlm.nih.gov/Blast.cgi?PAGE_TYPE=BlastSearch%26;PROG_DEF=blastn%26;BLAST_SPEC=GDH_GCA_947034695.1. Accessed 25 Oct 2024.

[33] Garrison E, Guarracino A, Heumos S, Villani F, Bao Z, Tattini L, et al. Building pangenome graphs. Nat Methods. 2024;21:2008–12. 39433878 10.1038/s41592-024-02430-3

[34] Wick RR, Schultz MB, Zobel J, Holt KE. Bandage: interactive visualization of de novo genome assemblies. Bioinformatics. 2015;31(20):3350–2. 26099265 10.1093/bioinformatics/btv383 PMC4595904

[35] Ensembl - Biomart. https://www.ensembl.org/biomart/martview. Accessed 9 Sep 2024.

[36] UCSC Genome Browser Gateway. https://genome-euro.ucsc.edu/cgi-bin/hgGateway?redirect=manual&source=genome.ucsc.edu. Accessed 9 Sep 2024.

[37] Salavati M, Clark R, Becker D, Kühn C, Plastow G, Dupont S, et al. Improving the annotation of the cattle genome by annotating transcription start sites in a diverse set of tissues and populations using cap analysis gene expression sequencing. G3 Genes|Genomes|Genetics. 2023;13:jkad108. 37216666 10.1093/g3journal/jkad108 PMC10411599

[38] Yuan C, Tang L, Lopdell T, Petrov VA, Oget-Ebrad C, Moreira GCM, et al. An organism-wide ATAC-seq peak catalog for the bovine and its use to identify regulatory variants. Genome Res. 2023;33:1848–64. 37751945 10.1101/gr.277947.123 PMC10691486

[39] Marakulina D, Vorontsov IE, Kulakovskiy IV, Lennartsson A, Drabløs F, Medvedeva YA. Epifactors 2022: expansion and enhancement of a curated database of human epigenetic factors and complexes. Nucleic Acids Res. 2023;51:D564-70. 36350659 10.1093/nar/gkac989 PMC9825597

[40] Castro-Mondragon JA, Riudavets-Puig R, Rauluseviciute I, Berhanu Lemma R, Turchi L, Blanc-Mathieu R, et al. JASPAR 2022: the 9th release of the open-access database of transcription factor binding profiles. Nucleic Acids Res. 2022;50:D165–73. 34850907 10.1093/nar/gkab1113 PMC8728201

[41] Bailey TL, Grant CE. SEA: Simple Enrichment Analysis of motifs. BioRxiv. 2021;2021(08):23.457422. 10.1101/2021.08.23.457422.

[42] Powers NR, Parvanov ED, Baker CL, Walker M, Petkov PM, Paigen K. The meiotic recombination activator PRDM9 trimethylates both H3K36 and H3K4 at recombination hotspots in vivo. PLoS Genet. 2016;12:e1006146. 27362481 10.1371/journal.pgen.1006146 PMC4928815

[43] Zoch A, Auchynnikava T, Berrens RV, Kabayama Y, Schöpp T, Heep M, et al. SPOCD1 is an essential executor of piRNA-directed de novo DNA methylation. Nature. 2020;584:635–9. 32674113 10.1038/s41586-020-2557-5 PMC7612247

[44] Malonia SK, Sinha S, Lakshminarasimhan P, Singh K, Jalota-Badhwar A, Rampalli S, et al. Gene regulation by SMAR1: role in cellular homeostasis and cancer. Biochimica et Biophysica Acta (BBA). 2011;1815:1–12. 20709157 10.1016/j.bbcan.2010.08.003

[45] Grand RS, Burger L, Gräwe C, Michael AK, Isbel L, Hess D, et al. BANP opens chromatin and activates CpG-island-regulated genes. Nature. 2021;596:133–7. 34234345 10.1038/s41586-021-03689-8

[46] Nakka KK, Chaudhary N, Joshi S, Bhat J, Singh K, Chatterjee S, et al. Nuclear matrix-associated protein SMAR1 regulates alternative splicing via HDAC6-mediated deacetylation of Sam68. Proc Natl Acad Sci U S A. 2015;112:E3374-83. 26080397 10.1073/pnas.1418603112 PMC4491761

[47] Alam A, Taye N, Patel S, Thube M, Mullick J, Shah VK, et al. SMAR1 favors immunosurveillance of cancer cells by modulating calnexin and MHC I expression. Neoplasia. 2019;21:945–62. 31422285 10.1016/j.neo.2019.07.002 PMC6706529

[48] Palamarchuk A, Yan PS, Zanesi N, Wang L, Rodrigues B, Murphy M, et al. Tcl1 protein functions as an inhibitor of de novo DNA methylation in B-cell chronic lymphocytic leukemia (CLL). Proc Natl Acad Sci U S A. 2012;109:2555–60. 22308499 10.1073/pnas.1200003109 PMC3289317

[49] Grundberg E, Meduri E, Sandling JK, Hedman AK, Keildson S, Buil A, et al. Global analysis of DNA methylation variation in adipose tissue from twins reveals links to disease-associated variants in distal regulatory elements. Am J Hum Genet. 2013;93:876–90. 24183450 10.1016/j.ajhg.2013.10.004 PMC3824131

[50] Gaunt TR, Shihab HA, Hemani G, Min JL, Woodward G, Lyttleton O, et al. Systematic identification of genetic influences on methylation across the human life course. Genome Biol. 2016;17:61. 27036880 10.1186/s13059-016-0926-z PMC4818469

[51] Slieker RC, Roost MS, van Iperen L, Suchiman HED, Tobi EW, Carlotti F, et al. DNA methylation landscapes of human fetal development. PLoS Genet. 2015;11: e1005583. 26492326 10.1371/journal.pgen.1005583 PMC4619663

[52] Smith AK, Kilaru V, Kocak M, Almli LM, Mercer KB, Ressler KJ, et al. Methylation quantitative trait loci (meQTLs) are consistently detected across ancestry, developmental stage, and tissue type. BMC Genomics. 2014;15: 145. 24555763 10.1186/1471-2164-15-145 PMC4028873

[53] Lin D, Chen J, Perrone-Bizzozero N, Bustillo JR, Du Y, Calhoun VD, et al. Characterization of cross-tissue genetic-epigenetic effects and their patterns in schizophrenia. Genome Med. 2018;10: 13. 29482655 10.1186/s13073-018-0519-4 PMC5828480

[54] Feinberg JI, Schrott R, Ladd-Acosta C, Newschaffer CJ, Hertz-Picciotto I, Croen LA, et al. Epigenetic changes in sperm are associated with paternal and child quantitative autistic traits in an autism-enriched cohort. Mol Psychiatry. 2024;29:43–53. 37100868 10.1038/s41380-023-02046-7 PMC12450096

[55] Hop PJ, Luijk R, Daxinger L, Van Iterson M, Dekkers KF, Jansen R, et al. Genome-wide identification of genes regulating DNA methylation using genetic anchors for causal inference. Genome Biol. 2020;21: 220. 32859263 10.1186/s13059-020-02114-z PMC7453518

[56] Banovich NE, Lan X, McVicker G, Geijn B, van de, Degner JF, Blischak JD, et al. Methylation QTLs are associated with coordinated changes in transcription factor binding, histone modifications, and gene expression levels. PLoS Genet. 2014;10:e1004663. 25233095 10.1371/journal.pgen.1004663 PMC4169251

[57] Cedar H, Bergman Y. Linking DNA methylation and histone modification: patterns and paradigms. Nat Rev Genet. 2009;10:295–304. 19308066 10.1038/nrg2540

[58] Kiefer H, Perrier J-P. DNA methylation in bull spermatozoa: evolutionary impacts, interindividual variability, and contribution to the embryo. Can J Anim Sci. 2020;100:1–16.

[59] Sandor C, Li W, Coppieters W, Druet T, Charlier C, Georges M. Genetic variants in REC8, RNF212, and PRDM9 influence male recombination in cattle. PLoS Genet. 2012;8: e1002854. 22844258 10.1371/journal.pgen.1002854 PMC3406008

[60] Leonard AS, Mapel XM, Pausch H. Pangenome-genotyped structural variation improves molecular phenotype mapping in cattle. Genome Res. 2024;34:300–9. 38355307 10.1101/gr.278267.123 PMC10984387

[61] Attree E, Griffiths B, Panchal K, Xia D, Werling D, Banos G, et al. Identification of DNA methylation markers for age and bovine respiratory disease in dairy cattle: a pilot study based on reduced representation bisulfite sequencing. Commun Biol. 2024;7:1–15. 39363014 10.1038/s42003-024-06925-9 PMC11450024

[62] Dong W, Yang J, Zhang Y, Liu S, Ning C, Ding X, et al. Integrative analysis of genome-wide DNA methylation and gene expression profiles reveals important epigenetic genes related to milk production traits in dairy cattle. J Anim Breed Genet. 2021;138:562–73. 33620112 10.1111/jbg.12530

[63] Meissner A, Mikkelsen TS, Gu H, Wernig M, Hanna J, Sivachenko A, et al. Genome-scale DNA methylation maps of pluripotent and differentiated cells. Nature. 2008;454:766–70. 18600261 10.1038/nature07107 PMC2896277

[64] Göring HHH, Terwilliger JD, Blangero J. Large upward bias in estimation of locus-specific effects from genomewide scans. Am J Hum Genet. 2001;69:1357–69. 11593451 10.1086/324471 PMC1235546

