# Supplementary material for: Genetic regulation of sperm DNA methylation in cattle through meQTL mapping
Source: BMC Genomics. 2025 Aug 22;26:771. doi: 10.1186/s12864-025-11934-x (PMC12374274; doi:10.1186/s12864-025-11934-x)
Supplement: Supplementary file 1 — Supplementary Material 1. [file 12864_2025_11934_MOESM1_ESM.pdf]

Genetic regulation of sperm DNA methylation in cattle through meQTL mapping

Corentin Fouéré[1,2*], Valentin Costes[1,3,4], Chris Hozé[1,2], Amrita Raja Ravi Shankar[3,4], Florian Besnard[1,2], Gabriel Costa Monteiro Moreira[3,4], Valentin Sorin[2], Chrystelle Le Danvic[1,3,4], Aurélie Chaulot-Talmon[3,4], Francesca Ali[3,4], Marie Christine Deloche[1,3,4], Aurélie Bonnet[1,3,4], Eliaou Sellem[3,4], Hélène Jammes[3,4], Sébastien Fritz[1,2], Mekki Boussaha[2], Didier Boichard[2], Hélène Kiefer[3,4], Marie-Pierre Sanchez[2*]

[1] Eliance, 149 Rue de Bercy, 75012 Paris, France,

[2] Université Paris-Saclay, INRAE, AgroParisTech, GABI, 78352 Jouy-en-Josas, France,

[3] Université Paris-Saclay, UVSQ, INRAE, BREED, 78350, Jouy-en-Josas, France,

[4] Ecole Nationale Vétérinaire d'Alfort, BREED, 94700, Maisons-Alfort, France.

* Corresponding authors

Corentin Fouéré: corentin.fouere@inrae.fr

Marie-Pierre Sanchez: marie-pierre.sanchez@inrae.fr

Supplementary figures

Supplementary Figure 1

Supplementary Figure 2

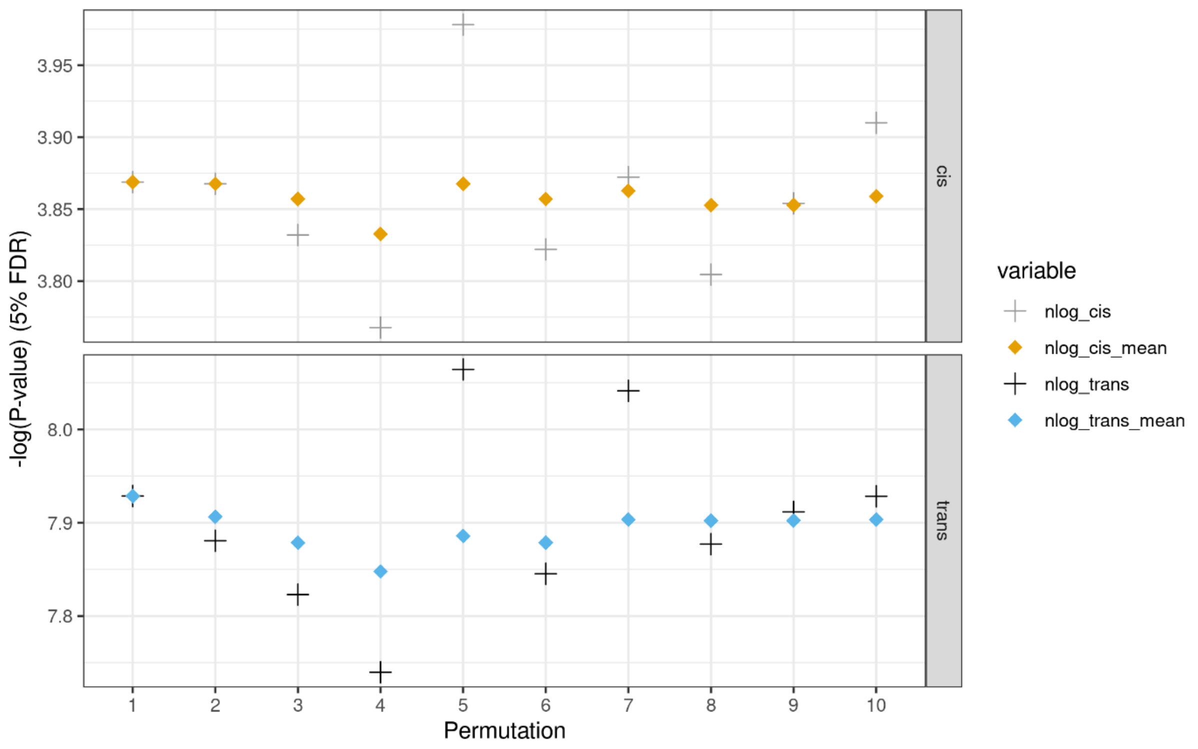

Supplementary Fig. 1 Result of permutations to account for multiple testing. Each plot represents, for each i permutation (i ranging from 1 to 10), the threshold calculated for the ith permutation (5% false positive rate, symbolised by the “+” sign) and the threshold averaged over the previous permutations, including permutation i (symbolised by the diamonds). Results for '*cis*' threshold at top and results for '*trans*' threshold at bottom.

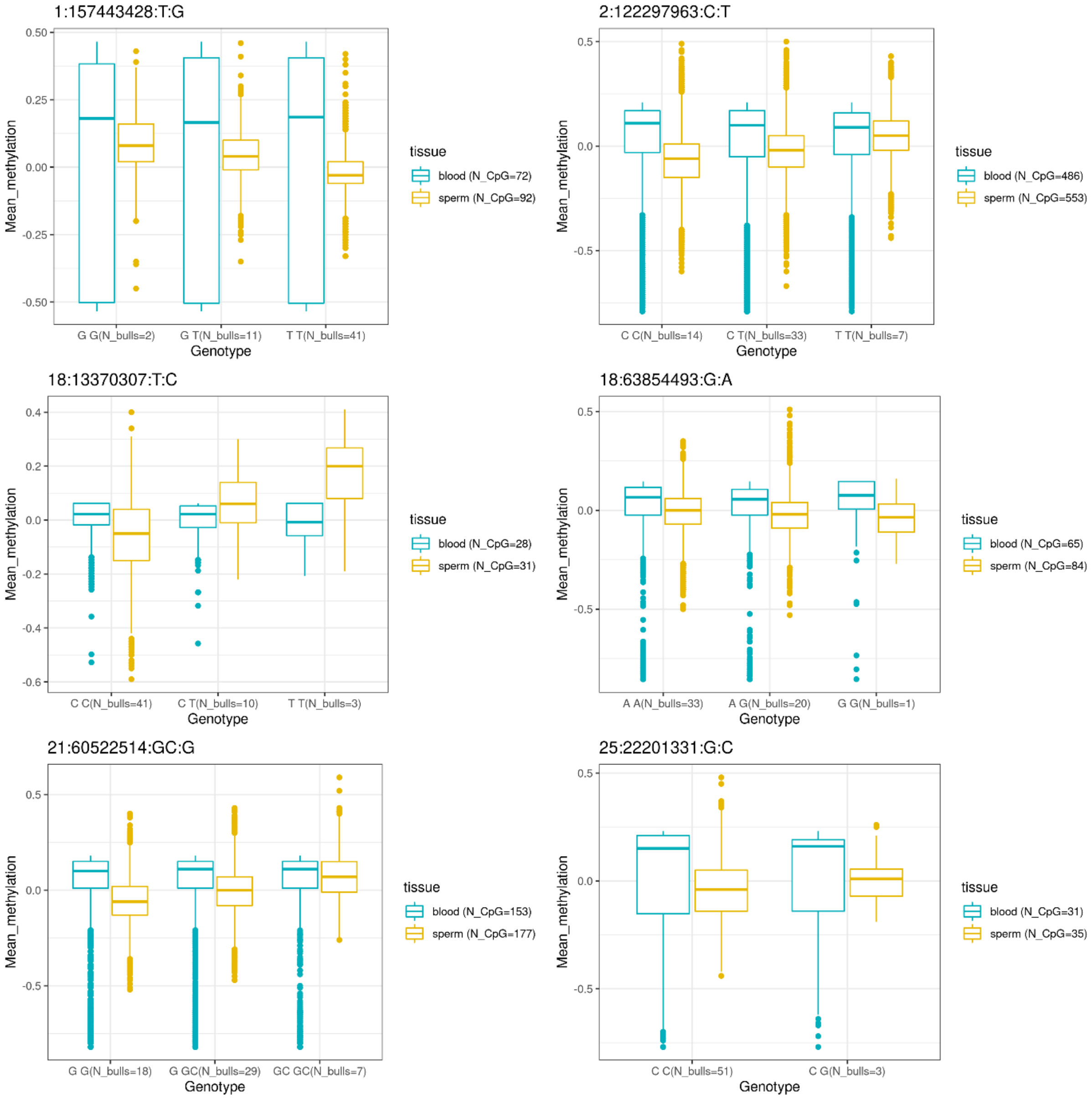

Supplementary Fig. 2 Methylation of *trans*-CpGs in PBMCs and sperm depending on the bull genotype. Each boxplot represents the CpGs associated with the lead SNP of 6 *trans*-meQTL hotspot for 54 bulls with DNAm information available for PBMCs and sperm. The 54 bulls were included for the meQTL mapping of sperm meQTLs. The number of bulls is indicated for each genotype. Mean methylation was centered on 0 for each *trans*-CpG group for each tissue.
